# Supplementary material for: Sec7 regulatory domains scaffold autoinhibited and active conformations
Source: bioRxiv. 2023 Nov 22:2023.11.22.568272. Preprint. [Version 1] doi: 10.1101/2023.11.22.568272 (PMC10690275; doi:10.1101/2023.11.22.568272)
Supplement: 1 [file NIHPP2023.11.22.568272v1-supplement-1.pdf]

bioRxiv preprint doi: https://doi.org/10.1101/2023.11.22.568272; this version posted November 22, 2023. The copyright holder for this preprint (which was not certified by peer review) is the author/funder, who has granted bioRxiv a license to display the preprint in perpetuity. It is made available under aCC-BY 4.0 International license.

SI Appendix

Sec7 regulatory domains scaffold autoinhibited and active conformations

Bryce A. Brownfield, Brian C. Richardson, Steve Halaby, and J. Christopher Fromme

bioRxiv preprint doi: https://doi.org/10.1101/2023.11.22.568272; this version posted November 22, 2023. The copyright holder for this preprint (which was not certified by peer review) is the author/funder, who has granted bioRxiv a license to display the preprint in perpetuity. It is made available under aCC-BY 4.0 International license.

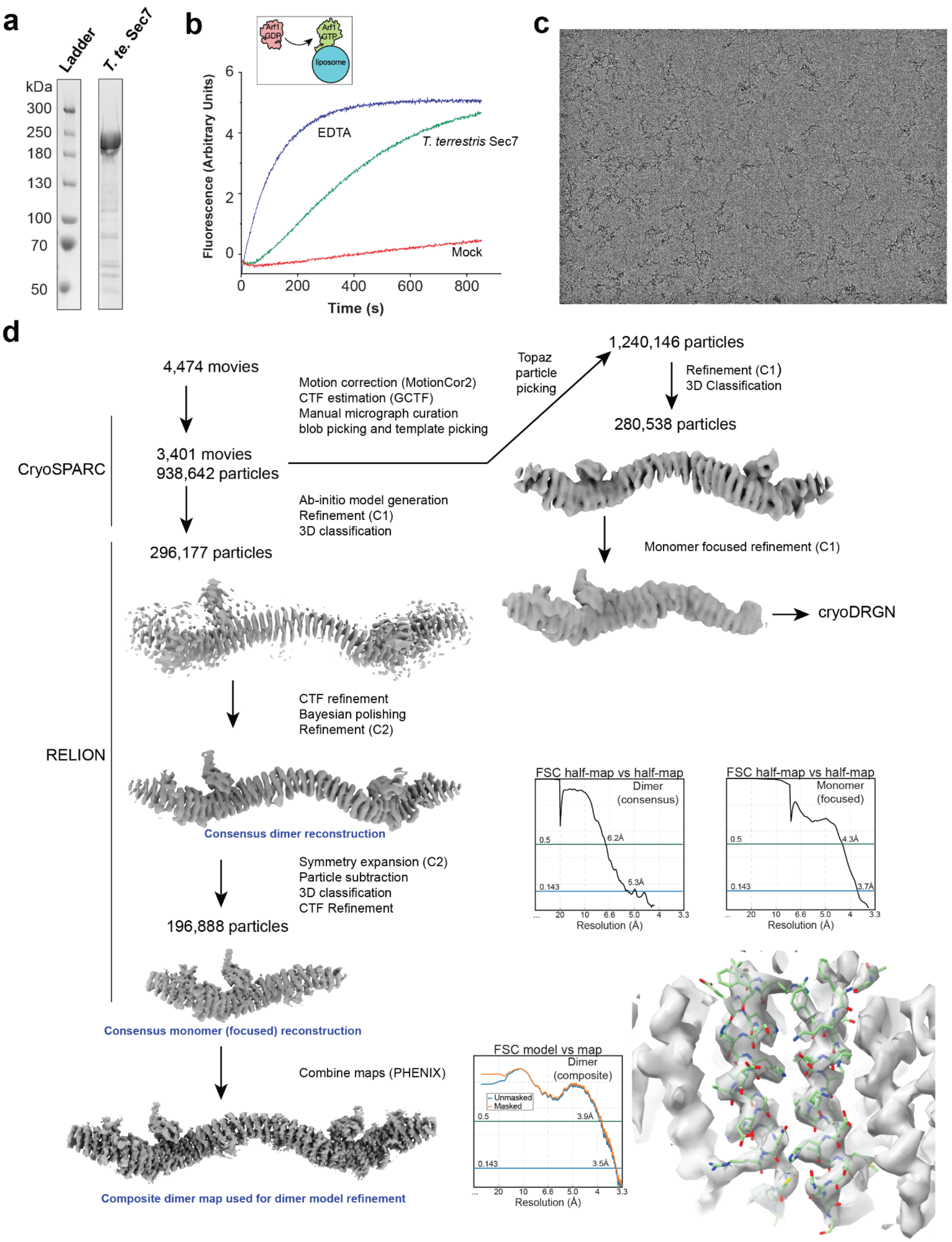

SI Appendix, Figure S1 - Legend on following page

bioRxiv preprint doi: https://doi.org/10.1101/2023.11.22.568272; this version posted November 22, 2023. The copyright holder for this preprint (which was not certified by peer review) is the author/funder, who has granted bioRxiv a license to display the preprint in perpetuity. It is made available under aCC-BY 4.0 International license.

SI Appendix, Figure S1 – *T. terrestris* Sec7 purification and CryoEM data processing workflow. a, SDS-PAGE of purified *T. terrestris* Sec7 expressed in *P. pastoris*. **b**, Tryptophan fluorescence GEF activity assay demonstrating that the purified *T. terrestris* Sec7 construct is capable of myristoylated-Arf1 activation in the presence of TGN liposomes (see Methods). Note that the observed rate and sigmoidal shape of the curve are consistent with autoinhibitory behavior previously determined for *S. cerevisiae* Sec7 (1). **c**, Representative cryoEM micrograph. **d**, CryoEM data processing workflow.

bioRxiv preprint doi: https://doi.org/10.1101/2023.11.22.568272; this version posted November 22, 2023. The copyright holder for this preprint (which was not certified by peer review) is the author/funder, who has granted bioRxiv a license to display the preprint in perpetuity. It is made available under aCC-BY 4.0 International license.

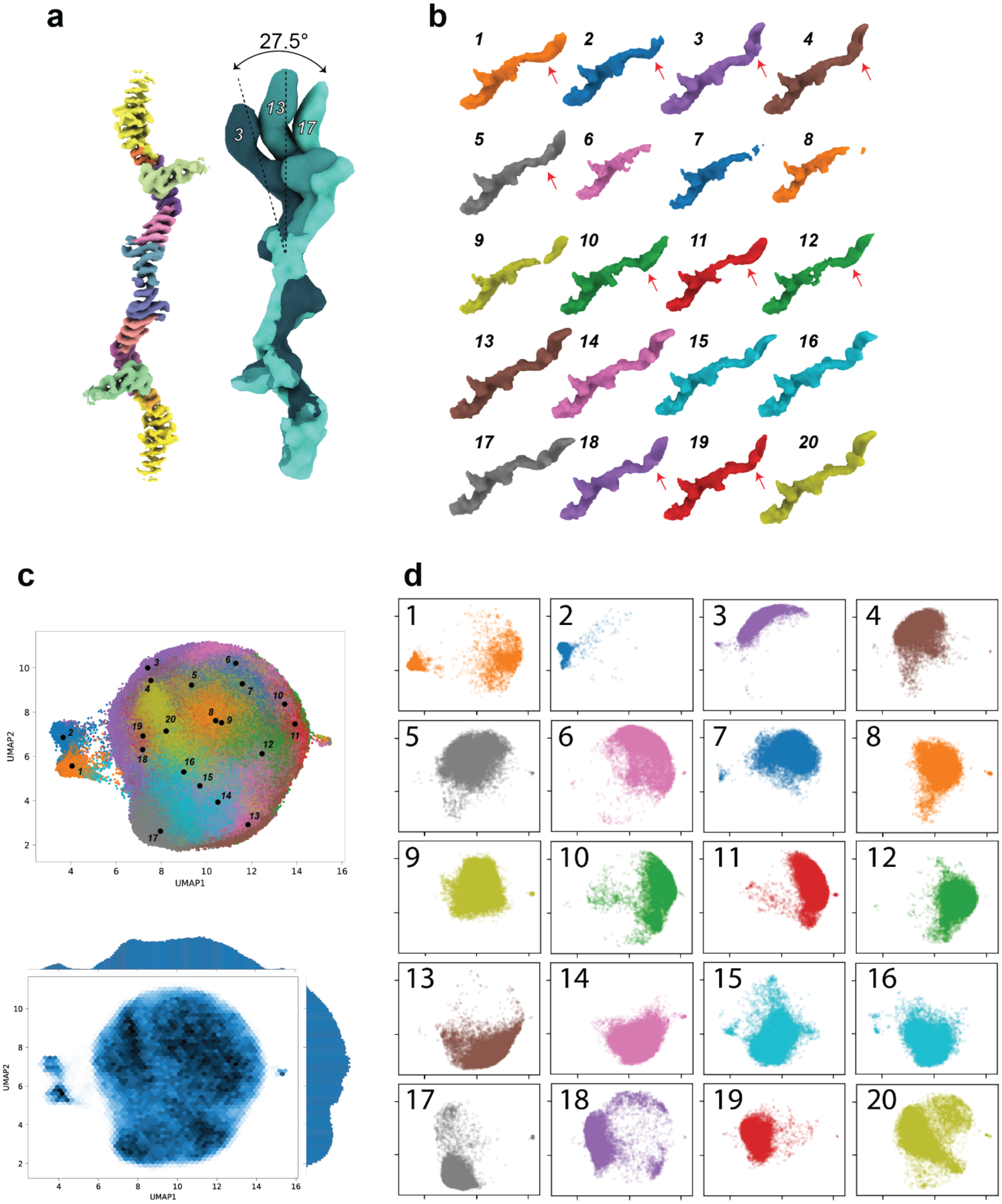

SI Appendix, Figure S2 – Heterogeneous Reconstruction and UMAP analysis by cryoDRGN. Dimer particles were focus-refined on a monomer (without symmetry expansion or subtraction) and used to train a cryoDRGN variability model (2). **a**, Cryo EM map of the Sec7 dimer reconstruction (left), and three example cryoDRGN generated maps highlighting the flexibility of the Sec7 dimer. **b**, All maps generated by cryoDRGN. Maps 6-10 significantly lack density for a second monomer, perhaps due to flexibility. Red arrows indicate maps with little/no density for the GEF domain bound to HDS2. We note that this only occurs in the monomer that was not focused on during refinement, and therefore could be artifactual. **c**, Plots of UMAP analysis colored by reconstruction weight of the maps in b (top) or by density of particles (bottom). **d**, Isolated UMAP distribution plots of each map in b.

bioRxiv preprint doi: https://doi.org/10.1101/2023.11.22.568272; this version posted November 22, 2023. The copyright holder for this preprint (which was not certified by peer review) is the author/funder, who has granted bioRxiv a license to display the preprint in perpetuity. It is made available under aCC-BY 4.0 International license.

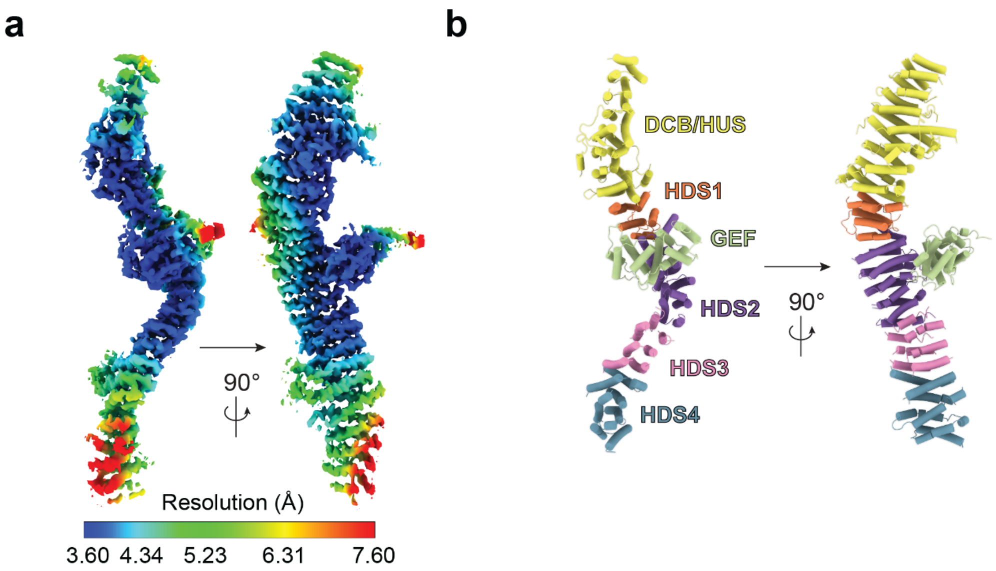

SI Appendix, Figure S3 – Focused refinement of a Sec7 monomer. a, Map generated by focused refinement of an entire single monomer using symmetry expanded particles gave the highest resolution, 3.7 Å overall. Local resolution determined using RELION (3) was used to color the map as indicated. **b**, Model built and refined using the map in a, colored by domains as indicated.

bioRxiv preprint doi: https://doi.org/10.1101/2023.11.22.568272; this version posted November 22, 2023. The copyright holder for this preprint (which was not certified by peer review) is the author/funder, who has granted bioRxiv a license to display the preprint in perpetuity. It is made available under aCC-BY 4.0 International license.

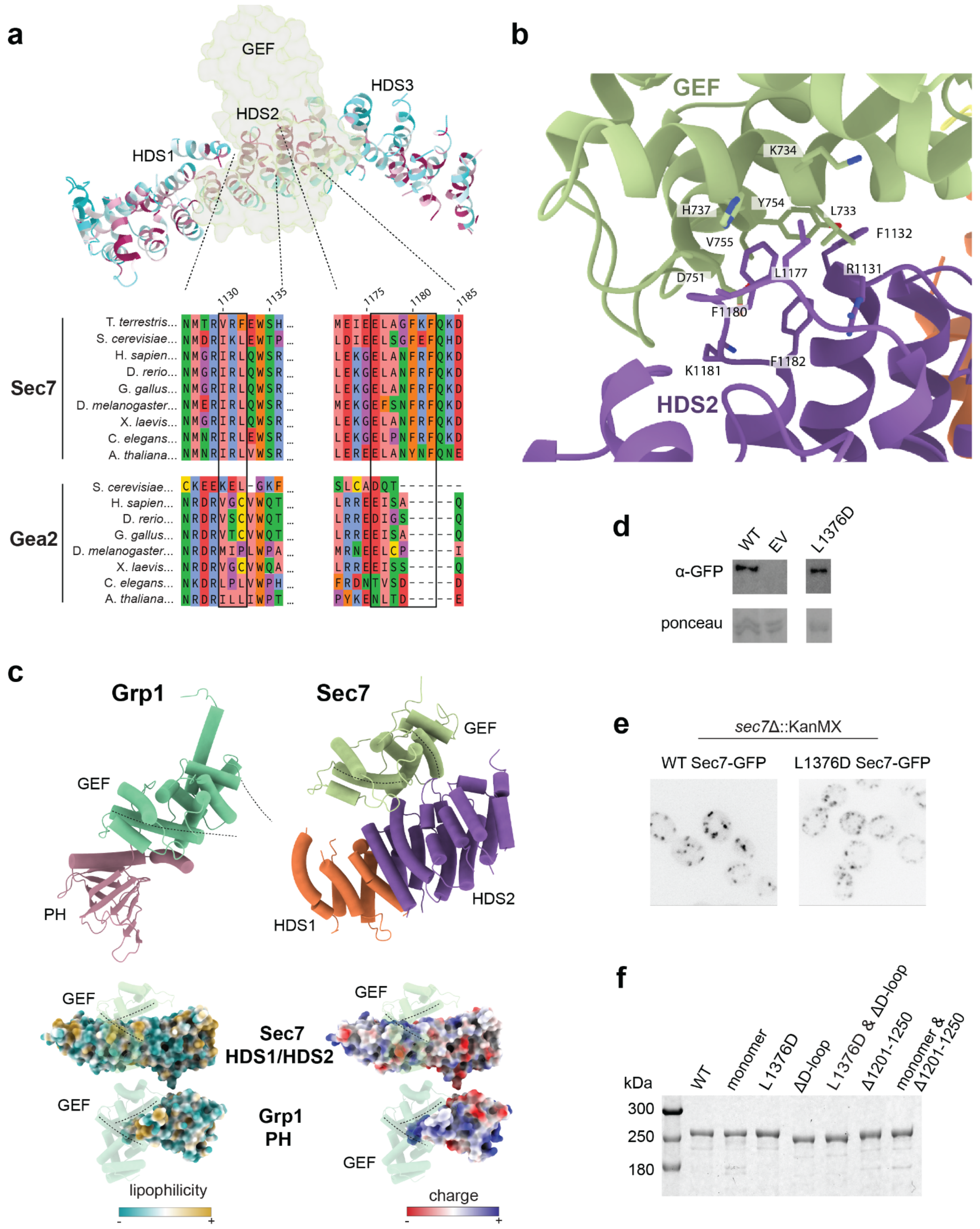

SI Appendix, Figure S4 – Legend on following page

bioRxiv preprint doi: https://doi.org/10.1101/2023.11.22.568272; this version posted November 22, 2023. The copyright holder for this preprint (which was not certified by peer review) is the author/funder, who has granted bioRxiv a license to display the preprint in perpetuity. It is made available under aCC-BY 4.0 International license.

SI Appendix, Figure S4 – Detailed view of HDS2 interface. a, Multiple sequence alignment of the HDS2 interface in Sec7 and Gea2 homologs. Boxed regions indicate the surface exposed loops that contact the GEF domain. **b**, Key residues of the HDS2 interface are shown. **c**, Comparison with the autoinhibited Grp1 crystal structure. Grp1 autoinhibition involves a basic patch in the PH domain that interacts with its GEF domain in a distinct manner from that of the Sec7 HDS2-GEF domain interaction. **d**, Western blot for α-GFP-Sec7, showing the L1376D construct is expressed similarly to WT after shuffling. **e**, Microscopy of GFP-Sec7 constructs after shuffling in *sec7*Δ::KanMX strain background. **f**, Example SDS-PAGE gel of purified protein constructs used in assays shown in Fig. 2d,e and Fig. 4a-d.

bioRxiv preprint doi: https://doi.org/10.1101/2023.11.22.568272; this version posted November 22, 2023. The copyright holder for this preprint (which was not certified by peer review) is the author/funder, who has granted bioRxiv a license to display the preprint in perpetuity. It is made available under aCC-BY 4.0 International license.

a

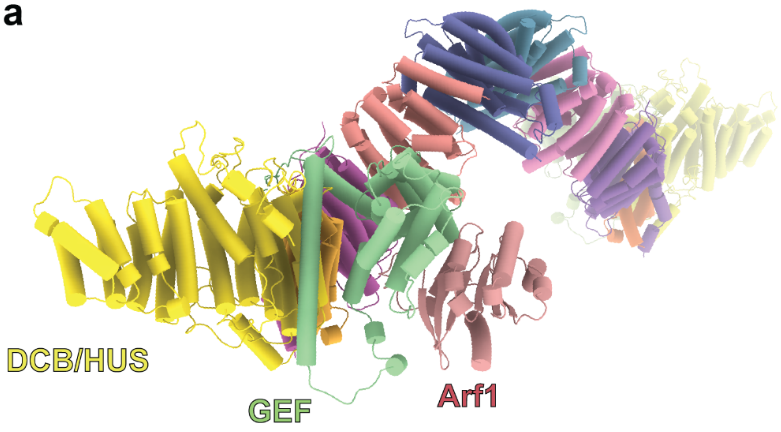

b

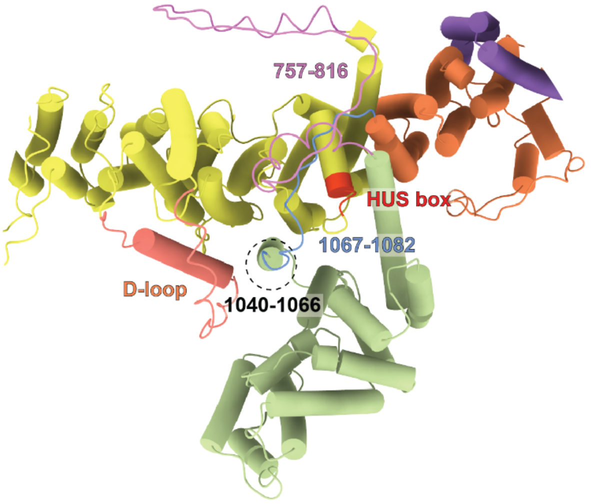

SI Appendix, Figure S5 – Predicted structural model of Sec7 in an active conformation. a, AlphaFold prediction with Arf1 bound to the GEF domain as in the Gea2-Arf1 cryoEM structure (4) shows the catalytic surface of the GEF domain is accessible in this conformation. **b**, Positions of the GEF linkers (757-816 and 1067-1082 in S. cerevisiae) surround the HUS box in the AlphaFold prediction, and physically connect the GEF domain to the activating surface of the DCB/HUS domains. Note residues 1040-1082 correspond to the GEF-HDS1 linker identified in the Gea2 cryoEM structure (4).

bioRxiv preprint doi: https://doi.org/10.1101/2023.11.22.568272; this version posted November 22, 2023. The copyright holder for this preprint (which was not certified by peer review) is the author/funder, who has granted bioRxiv a license to display the preprint in perpetuity. It is made available under aCC-BY 4.0 International license.

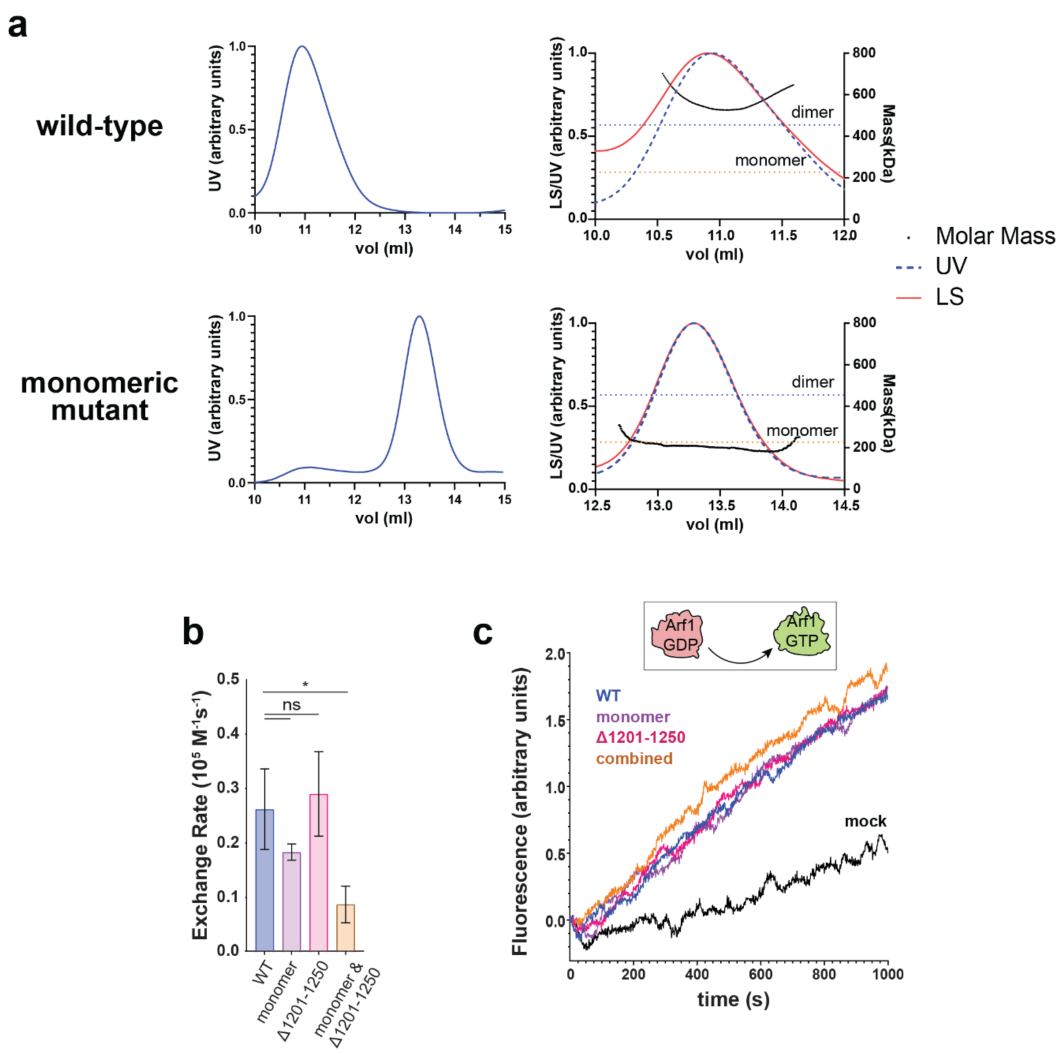

SI Appendix, Figure S6 – SEC/MALS of monomeric Sec7 construct and membrane binding. a, Purified monomeric Sec7 eluted ~2.5 ml later than WT Sec7 over a Superose 6 10/300 column (left), and was verified by MALS to be monomeric (right). **b**, Quantification of reaction rate constants for triplicate measurements as reported in Fig. 5g. n.s.: not significant, * $p < 0.05$ **c**, Representative traces of ΔN17-Arf1 exchange catalyzed by the indicated GEF constructs.

bioRxiv preprint doi: https://doi.org/10.1101/2023.11.22.568272; this version posted November 22, 2023. The copyright holder for this preprint (which was not certified by peer review) is the author/funder, who has granted bioRxiv a license to display the preprint in perpetuity. It is made available under aCC-BY 4.0 International license.

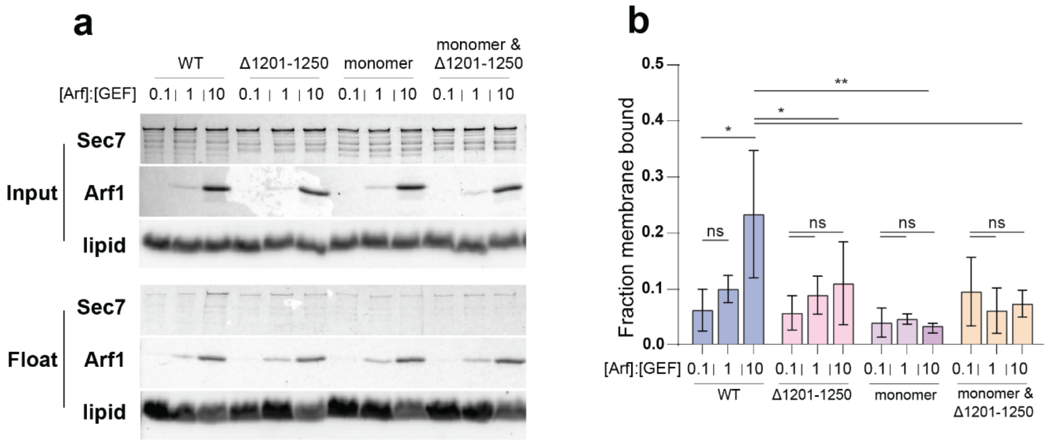

SI Appendix, Figure S7 – Dimerization and membrane binding. a, Representative SDS-PAGE of membrane float assay. **b**, Quantification of triplicate measurements in a. n.s.: not significant, * $p < 0.05$, * $p < 0.01$

bioRxiv preprint doi: https://doi.org/10.1101/2023.11.22.568272; this version posted November 22, 2023. The copyright holder for this preprint (which was not certified by peer review) is the author/funder, who has granted bioRxiv a license to display the preprint in perpetuity. It is made available under aCC-BY 4.0 International license.

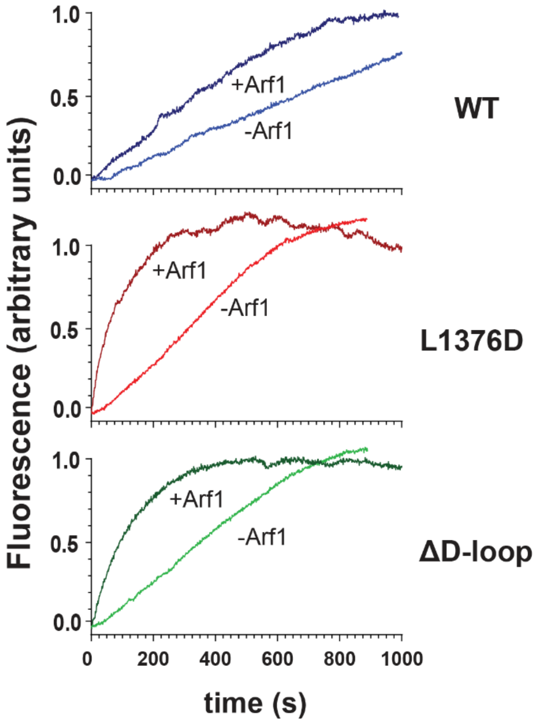

SI Appendix, Figure S8 – Raw data for Fig. 6d. Representative trace of GEF assay shown in Fig 6d.

bioRxiv preprint doi: https://doi.org/10.1101/2023.11.22.568272; this version posted November 22, 2023. The copyright holder for this preprint (which was not certified by peer review) is the author/funder, who has granted bioRxiv a license to display the preprint in perpetuity. It is made available under aCC-BY 4.0 International license.

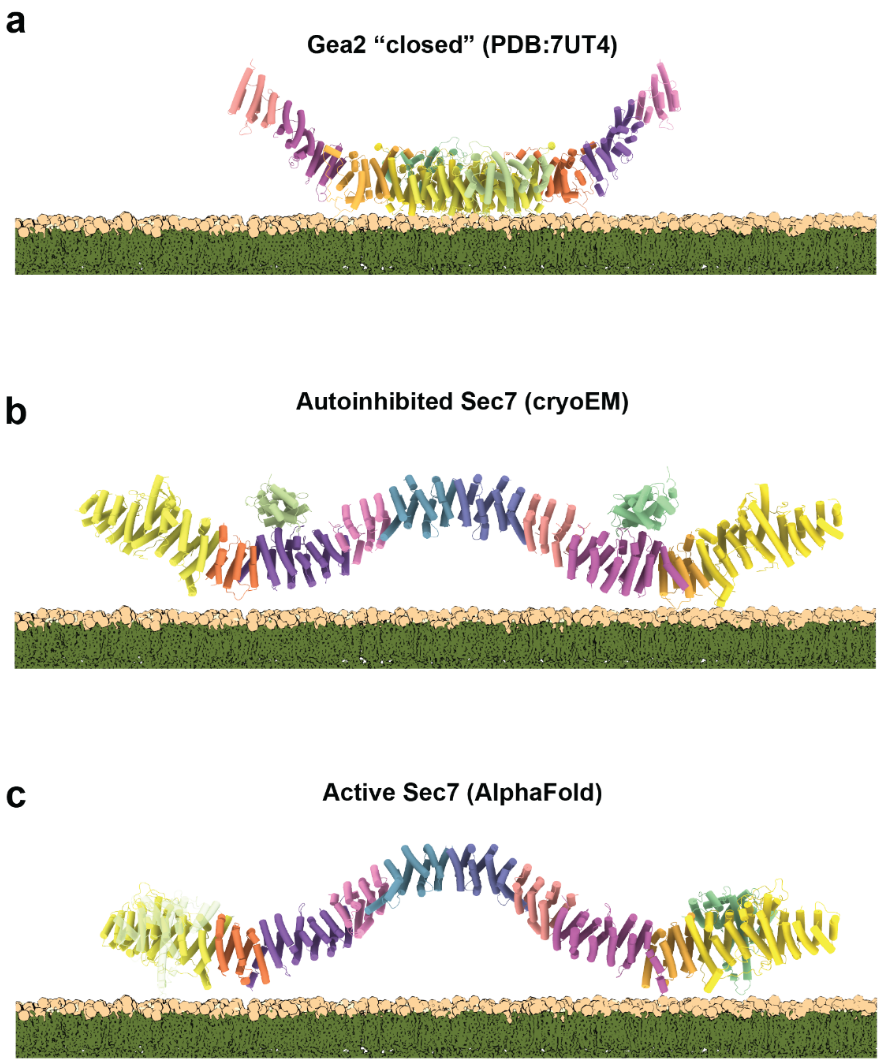

SI Appendix, Figure S9 – The autoinhibited conformation of Sec7 is not compatible with stable membrane binding. Comparison of Gea2 cryoEM (**a**), autoinhibited Sec7 cryoEM (**b**), and AlphaFold-predicted Sec7 (**c**) structures on the membrane surface.

bioRxiv preprint doi: https://doi.org/10.1101/2023.11.22.568272; this version posted November 22, 2023. The copyright holder for this preprint (which was not certified by peer review) is the author/funder, who has granted bioRxiv a license to display the preprint in perpetuity. It is made available under aCC-BY 4.0 International license.

SI Appendix, Table S1 – Plasmids

Name	Description*	Vector Backbone	Source
pPICZB	*P. pastoris* integration plasmid		Thermo Fischer Cat # V19020
	pPICZB with N terminal 12xHis-GFP-HrV 3C	pPICZB	This Study
pBB01271	pPICZB *T. terrestris* Sec7 (6x-His-Sec7-6xHis)	pPICZB	This Study
pBB01793	pPICZB *S. cerevisiae* Sec7 WT (12xHis-GFP-HR3 VC-Sec7)	pPICZB	This Study
pBB01921	pPICZB *S. cerevisiae* Sec7 monomer (12xHis-GFP-HR3 VC-Sec7 Y1975S, I1979S, L1982S, V1986S, L1998S, V2001S)	pPICZB	This Study
pBB01922	pPICZB *S. cerevisiae* Sec7 L1376D (12xHis-GFP-HR3 VC-Sec7 L1376D)	pPICZB	This Study
pBB01923	pPICZB *S. cerevisiae* Sec7 ΔD-loop (12xHis-GFP-HR3 VC-Sec7 Δ444-486)	pPICZB	This Study
pBB01924	pPICZB *S. cerevisiae* Sec7 L1376 & ΔD-loop (12xHis-GFP-HR3 VC-Sec7 L1376, Δ444-486)	pPICZB	This Study
pBB01925	pPICZB *S. cerevisiae* Sec7 Δ1201-1250 (12xHis-GFP-HR3 VC-Sec7 Δ1201-1250)	pPICZB	This Study
pBB01926	pPICZB *S. cerevisiae* Sec7 monomer & Δ1201-1250 (12xHis-GFP-HR3 VC-Sec7 Δ1201-1250, Y1975S, I1979S, L1982S, V1986S, L1998S, V2001S)	pPICZB	This Study
pRS415	pRS415 vector (centromeric LEU2 plasmid)		(5)
pCF1084	pRS415;GFP-Sec7		(1)
pBB01602	pRS415;GFP-Sec7 L1376D		This Study
pBB01761	pRS415;GFP-Sec7 L1376K		This Study
pBB01762	pRS415;GFP-Sec7 L1376S		This Study
pBB01871	pRS415;GFP-Sec7 ΔD-loop (Δ444-486)		This Study
pBB01863	pRS415;GFP-Sec7 L1331S, L1376S, F1379S		This Study

bioRxiv preprint doi: https://doi.org/10.1101/2023.11.22.568272; this version posted November 22, 2023. The copyright holder for this preprint (which was not certified by peer review) is the author/funder, who has granted bioRxiv a license to display the preprint in perpetuity. It is made available under aCC-BY 4.0 International license.

pBB01874	pRS415;GFP-Sec7 ΔD-loop (Δ444-486) & L1376D		This Study
pBB01876	pRS415;GFP-Sec7 ΔD-loop (Δ444-486) & L1331S, L1376S, F1379S		This Study
pBB01691	pRS415;GFP-Sec7 monomer (Y1975S, I1979S, L1982S, V1986S, L1998S, V2001S)		This Study
pBB01753	pRS415;GFP-Sec7 Δ1201-1250		This Study
pBB01867	pRS415;GFP-Sec7 monomer & Δ1201-1250 (Δ1201-1250, Y1975S, I1979S, L1982S, V1986S, L1998S, V2001S)		This Study
pArf1	Bacterial Arf1 Expression plasmid	pET3C	(6)
pBB131	Bacterial Nmt1 expression plasmid	pCYC	(7)
pCF1053	Arf1ΔN17	pET28	(1)
pLT72	Full-length Ypt31 with cleavable N-terminal GST tag	pGEX-6P	(8)
pLT40	Gdi1 with cleavable N-terminal GST tag	pGEX-6P	(8)
pLT35	Mrs6 with cleavable N-terminal His 6 tag	pET28	(8)
pLT41	Bet2 with cleavable N-terminal His 6 tag and Bet4	pCDF-Duet-1	(8)

* All constructs encode full-length proteins unless otherwise noted.

bioRxiv preprint doi: https://doi.org/10.1101/2023.11.22.568272; this version posted November 22, 2023. The copyright holder for this preprint (which was not certified by peer review) is the author/funder, who has granted bioRxiv a license to display the preprint in perpetuity. It is made available under aCC-BY 4.0 International license.

SI Appendix, Table S2 – Bacterial and Yeast Strains

Name	Genotype	Source
Rosetta2, *E. coli*		Novagen Cat # 71400
DH5α, *E. coli*		New England Biolabs Cat # C2987I
BY4742	*MATα his3-Δ1 leu2-Δ0 lys2-Δ0 ura3-Δ0*	(9)
CFY409	BY4742 *sec7Δ::KANMX* + pCF1043	(1)
CFY863	CFY409 *arf1Δ::HIS3* + pCF1043	(1)
CFY4969	CFY409 *age2Δ::HIS3* + pCF1043	This study
KM71H (*P. pastoris*)		ThermoFisher Scientific Cat # C18200
CFY4970	KM71H *pAOX1*::6x-His-Sec7(*T. terrestris*)-6xHis::*BleoR*	This study
CFY4971	KM71H *pAOX1*::12xHis-GFP-HR3 VC-Sec7 (*S. cerevisiae*)::*BleoR*	This study
CFY4972	KM71H *pAOX1*::12xHis-GFP-HR3 VC-Sec7 monomer (*S. cerevisiae*, Y1975S, I1979S, L1982S, V1986S, L1998S, V2001S)::*BleoR*	This study
CFY4973	KM71H *pAOX1*::12xHis-GFP-HR3 VC-Sec7 L1376D (*S. cerevisiae*)::*BleoR*	This study
CFY4974	KM71H *pAOX1*::12xHis-GFP-HR3 VC-Sec7 ΔD-loop (*S. cerevisiae*, Δ444-486)::*BleoR*	This study
CFY4975	KM71H *pAOX1*::12xHis-GFP-HR3 VC-Sec7 ΔD-loop & L1376D (*S. cerevisiae*, Δ444-486, L1376D)::*BleoR*	This study
CFY4976	KM71H *pAOX1*::12xHis-GFP-HR3 VC-Sec7 Δ1201-1250 (*S. cerevisiae*)::*BleoR*	This study
CFY4977	KM71H *pAOX1*::12xHis-GFP-HR3 VC-Sec7 monomer & Δ1201-1250 (*S. cerevisiae*, Δ1201-1250, Y1975S, I1979S, L1982S, V1986S, L1998S, V2001S)::*BleoR*	This study

bioRxiv preprint doi: https://doi.org/10.1101/2023.11.22.568272; this version posted November 22, 2023. The copyright holder for this preprint (which was not certified by peer review) is the author/funder, who has granted bioRxiv a license to display the preprint in perpetuity. It is made available under aCC-BY 4.0 International license.

SI Appendix, Table S3 - TGN lipid mix

Lipid	mol%
DOPC	24
POPC	6
DOPE	7
POPE	3
DOPS	1
POPS	2
DOPA	1
POPA	2
Liver or Soy PI	30
PI(4)P	1
CDP-DAG	2
PO-DAG	4
DO-DAG	2
Ceramide (C18)	5
Cholesterol	10
DiR	1

bioRxiv preprint doi: https://doi.org/10.1101/2023.11.22.568272; this version posted November 22, 2023. The copyright holder for this preprint (which was not certified by peer review) is the author/funder, who has granted bioRxiv a license to display the preprint in perpetuity. It is made available under aCC-BY 4.0 International license.

SI Appendix, Video Legends

SI Appendix, Video S1

Video of the Sec7 dimer cryoEM structure. The presumptive membrane-binding surface is at the bottom of the molecule.

SI Appendix, Video S2

Hypothetical model for the conformational changes Sec7 undergoes when switching from the autoinhibited to active states. A model of the inactive conformation containing all residues of *T. terrestris* Sec7 was generated by SWISS-MODEL (10) templated with the cryoEM experimental model, and then a morphing transition was created using the AlphaFold predicted model of *T. terrestris* Sec7 in ChimeraX (11). The first few frames of this morph are looped in the beginning to highlight the presumed spontaneous, but infrequent, dissociation of the GEF domain from the HDS2 domain. Once Sec7 adopts the active conformation, it can be stabilized by binding to the activated forms of regulatory GTPases such as Arl1 (depicted in blue), allowing activation of the Arf1 GTPase substrate (depicted in red).

bioRxiv preprint doi: https://doi.org/10.1101/2023.11.22.568272; this version posted November 22, 2023. The copyright holder for this preprint (which was not certified by peer review) is the author/funder, who has granted bioRxiv a license to display the preprint in perpetuity. It is made available under aCC-BY 4.0 International license.

Supplementary Methods

Cloning of expression and purification constructs

All fragments used for cloning (insert and vector) were generated by PCR (Q5 polymerase, NEB Cat. No. E0555). When the template DNA carried the same selection marker as the final construct, it was linearized by a restriction enzyme that cleaved outside the desired amplicon prior to PCR to limit background transformants, and assembled by Gibson assembly (NEB Cat. No. E2621). *S. cerevisiae* Sec7 constructs used for complementation tests (plasmid shuffling assay) were cloned into the pRS415 vector with the Sec7 promoter and terminator. All yeast constructs are full-length and N-terminally GFP tagged, and otherwise only modified as indicated.

Purification constructs were cloned into the pPICZB vector. The full-length *T. terrestris* construct used for cryoEM was cloned from a plasmid containing *T. terrestris* Sec7 cDNA our group generated in a previous study (12), subcloned into pPICZB with 3C protease-cleavable 6xHis tags on the N and C termini. The full-length *S. cerevisiae* constructs were cloned into the same vector, but with an N-terminal 12xHis-GFP-Hrv3 tag. All constructs were verified by sequencing.

Strain modification

Standard genetic techniques were used to generate yeast strains (SI Appendix, Table S2). To generate the *sec7*Δ*age2*Δ strain, BY4741 *sec7*Δ::KanMX (CFY409) was transformed with a pFa6 plasmid-templated (13) *age2Δ::HIS3* cassette using standard LiOAc transformation. Cells were grown to an OD of 0.6 in YPD, washed with water twice by centrifugation, then resuspended and incubated at RT in 100 mM Lithium acetate, 1 mM EDTA, 10 mM Tris pH 8.0 for 15 minutes (cells were concentrated ~50 fold). 50 ul of this cell mixture was then added to 250 ul of transformation mix (30.5% PEG 3350, 100 mM LiOAc, 0.226 mg/ml salmon sperm DNA, 0.5-1 ug DNA) and incubated for 30 minutes with gentle agitation (rotating). For genomic integrations, 2 mM DTT was included in the transformation mix. The cells/transformation mixture was then heat shocked at 42 °C for 15 min. For drug selection, cells were washed and recovered for 3 hours in YPD before plating. For auxotrophic selection, cells were washed once in YNB with 5% YPD supplemented and plated immediately. *Pichia pastoris* expression strains were generated by transforming the KM71H strain using electroporation as described previously (14) after linearizing the pPICZB-Sec7 cassette with PmeI.

bioRxiv preprint doi: https://doi.org/10.1101/2023.11.22.568272; this version posted November 22, 2023. The copyright holder for this preprint (which was not certified by peer review) is the author/funder, who has granted bioRxiv a license to display the preprint in perpetuity. It is made available under aCC-BY 4.0 International license.

Purification of *T. terrestris* Sec7 for cryoEM

After transformation, 5 colonies of CFY4970 were patched together on a fresh Zeocin plate, and cultured with autoinduction media as described(15). Cells were collected by centrifugation after 48hrs, and yeast cell paste was flash frozen in liquid nitrogen in small aliquots. Cell paste was then lysed in a Spex cryogenic mill (6875D) for 15 cycles, 15 cps, 2 min rest between cycles, and powder was stored at -80 until further processing. 20 g of lysate powder was thawed rapidly by adding 18 ml room temperature thaw buffer (55 mM Hepes, pH 7.4, 495 mM KOAc, 11% glycerol, 44 mM Imidazole, 1.1 mM DTT, 1.1 mM AEBSF, and 1.1 x Roche cOmplete Protease Inhibitor Cocktail) and sonicated with a macro-tip sonicator (100% power, 1s on 1s off) until mostly thawed (~2 min). Then, 2 ml of 10% CHAPS was added (final concentration of 0.5%) and further sonicated (20s) to solubilize membranes. This lysate was clarified by centrifugation (20 min, 10,000 rpm in AV10 rotor, and then 30 min 20,000 rpm in SS-34 rotor), and loaded onto a pre-equilibrated HisTrap column (Cytiva) with a syringe. Wash and elution was performed using an AKTA Pure with two buffers: Buffer A (50 mM Hepes, pH 7.4, 450 mM NaCl, 5% glycerol, 40 mM Imidazole, 1 mM DTT, and 0.1% CHAPS) and Buffer B (50 mM Hepes, pH 7.4, 300 mM NaCl, 10% glycerol, 500 mM Imidazole, 1 mM DTT, and 0.1% CHAPS). The column was washed with 25 CVs of 4% B, followed by a gradient to 35% B over 10 CVs to elute. Elution fractions were checked by SDS-PAGE, pooled, concentrated, flash frozen in liquid nitrogen, and stored at -80°C for later use.

Purification of *S. cerevisiae* proteins for biochemical analysis

S. cerevisiae Sec7 constructs were grown, induced, and harvested as described above. Batch affinity purification was performed with resin that had been fragmented by sonication (see below). The clarified lysate was incubated with the resin for 2 hours at 4 °C with rotation. After binding the resin was washed five times with 10 ml wash buffer (50 mM Hepes, pH 7.4, 450 mM NaCl, 5% glycerol, 40 mM Imidazole, 1 mM DTT, and 0.1% CHAPS), and transferred to a fresh tube after the third wash. Sec7 was eluted from the NiNTA resin by 3C protease cleavage overnight at 4 °C. This elution was further purified by size exclusion chromatography using a Sepharose 6 increase 10/300 (SEC buffer: 25 mM Hepes, pH 7.4, 250 mM NaCl, 5% glycerol, 1 mM DTT). SEC fractions were analyzed by SDS-PAGE, pooled, concentrated, flash frozen in liquid nitrogen, and stored at -80 for later use.

bioRxiv preprint doi: https://doi.org/10.1101/2023.11.22.568272; this version posted November 22, 2023. The copyright holder for this preprint (which was not certified by peer review) is the author/funder, who has granted bioRxiv a license to display the preprint in perpetuity. It is made available under aCC-BY 4.0 International license.

ΔN17-Arf1, myristoylated Arf1, and prenylated-Ypt31/GDI complex were purified as previously described(1, 8, 16).

Preparation of fragmented affinity resin

In order to increase the surface area of the Ni-NTA resin, the resin was resuspended in water to make a 20% slurry, then sonicated at 80% power for three minutes with a macro-tip, (cycles: 20s on / 10s off). Following sonication we equilibrated the fragmented resin 5x with buffer at a lower speed (1000 rpm) to remove resin fines.

CryoEM data processing

Movies were motion-corrected and dose-weighted using MotionCor2 (17), and micrograph defocus values were estimated using GCTF(18). Micrographs were manually inspected and culled to 3,401 usable micrographs, which were imported into CryoSPARC for particle picking (19). Defocus values were estimated with patch-CTF estimation in CryoSPARC and ‘blob picker’ was used to pick an initial set of particles. 2D-classification was used to generate templates for template picking. An initial set of 938,642 particles was used for Ab-initio model generation and 3D-classification was performed iteratively using heterogeneous 3D refinement to generate a final set of 296,177 particles.

These particles were re-extracted in RELION 3.1(20) and assigned GCTF-estimated per-particle defocus values. Iterative rounds of CTF refinement and Bayesian polishing improved the resolution from 7.14 Å to 5.3 Å. 3D classification was attempted, but no improvement was attainable for the dimer reconstruction. Focused refinement on a monomer(21) was performed using a mask including a single full monomer and a small portion of the other (corresponding approximately to the HDS4 domain) for particle subtraction after symmetry expansion. A second monomer mask containing only a single monomer was used for refinement. After several iterations of CTF refinement, fixed angle 3D classification isolated 196,888 symmetry expanded particles which produced a 3.7 Å (0.143 FSC) reconstruction after iterative CTF refinement. The published crystal structure of the DCB-HUS domain and AlphaFold prediction(12, 22) were used for guidance with *de novo* building in the few regions with poor side chain density. A monomeric atomic model was refined into the monomer map using Real Space Refine(23) in Phenix(24). A model for the dimer was then generated and

bioRxiv preprint doi: https://doi.org/10.1101/2023.11.22.568272; this version posted November 22, 2023. The copyright holder for this preprint (which was not certified by peer review) is the author/funder, who has granted bioRxiv a license to display the preprint in perpetuity. It is made available under aCC-BY 4.0 International license.

refined into a composite dimer map produced from the monomer map and the consensus dimer map using Phenix Combine Maps, and validated in Phenix(24–26). See Tables 1 and 2 and SI Appendix, Figures S1 and S3.

For cryoDRGN analysis, we used TOPAZ to increase the likelihood of rare particles(27) (this did not improve resolution of the monomer). Starting with 1,240,146 topaz picked particles heterogeneous 3D classification in CryoSPARC generated a final stack of 280,528 particles that were then used to generate a dimer map with C2 symmetry imposed (6.3 A), followed by a focused monomer refinement without subtraction before cryoDRGN training and analysis (SI Appendix, Figure S2) (2).

Fluorescence microscopy

Cells were grown overnight at 30 °C in liquid selection media (-Leu) to an OD of 0.6. Cells were allowed to settle on a coverslip dish (MatTek) for 10 min, and washed with fresh media. Imaging for SI Appendix, Figure S4 was done using a CSU-X spinning-disk confocal system (Intelligent Imaging Innovations) with a DMI6000 B microscope (Leica), 10031.46 NA oil immersion objective, and a QuantME EMCCD camera (Photometrics). Imaging was done using a DeltaVision Elite system equipped with an Olympus IX-71 inverted microscope, a DV Elite complementary metal-oxide semiconductor camera, a ×100/1.4 NA oil objective, and a DV Light SSI 7 Color illumination system with Live Cell Speed Option with DV Elite filter sets. Exposure and laser power were adjusted according to intensity, and were kept the same for all specimens being compared in an experiment. The brightness/intensity was equivalently adjusted across all images in an experiment using ImageJ.

Liposome preparation

Synthetic TGN liposomes were prepared as described previously(16). In brief – lipid stocks in chloroform were combined in a pear-shaped flask to produce a lipid mixture mimicking that of the yeast TGN(28) (SI Appendix, Table S3). Chloroform was evaporated slowly in a rotary evaporator heated to ~37 °C, then rehydrated in HK buffer (20mM HEPES pH 7.5, 150 mM KOAc) at 37 °C overnight. After gentle resuspension, the mixture was extruded through 100 nm filters 21 times and stored at 4 °C for no more than 1 month.

bioRxiv preprint doi: https://doi.org/10.1101/2023.11.22.568272; this version posted November 22, 2023. The copyright holder for this preprint (which was not certified by peer review) is the author/funder, who has granted bioRxiv a license to display the preprint in perpetuity. It is made available under aCC-BY 4.0 International license.

Liposome flotation membrane binding assay

Liposome flotation was performed as described(29). Briefly – 100 nm liposomes were loaded with Arf1 by EDTA exchange of GMPPNP for a final concentration of 250 µM lipid and the indicated Arf1 concentration. Sec7 constructs were added to a final concentration of 550 nM, and incubated at room temperature for 1 hour. Then 2.5 M sucrose in HK (20mM HEPES pH 7.5, 150 mM KOAc) was added to 1 M final concentration, and 80 µl was transferred to a polycarbonate tube. Then a 100 ul layer of 0.75 M Sucrose was added, followed by 20 µl of HK. This was centrifuged at 20 oC in a TLA100 rotor for 30 minutes, and the top layer collected for SDS-PAGE analysis (12% acrylamide gel).

In vitro Arf activation (GEF) assay

GEF activity was determined by measuring the native Tryptophan fluorescence of Arf1, as described previously(16). Briefly, to a final volume of 150 µl in HKM (20mM HEPES pH 7.5, 150 mM KOAc, 2 mM $MgCl_2$, 1 mM DTT), 200 µM 100 nm TGN liposomes, Sec7 construct (with concentration as detailed below), 1 µM myristoylated Arf1, and 200 µM GTP were added to a quartz cuvette. For Figures 2 and 4, GEF was added to a final concentration of 100 nM. For Figure 6b, 20 nM GEF was added. For Figure 6a, 60 nM GEF was added. For Figure 5g and SI Appendix, Figure S6c, 200 nM GEF was added. Tryptophan fluorescence (297.5 nm excitation, 340 nm emission) was measured using a fluorometer, and between additions the reaction was well mixed and the fluorescence was allowed to stabilize. The order listed is the order components were added, except for reactions with preloaded Ypt31. For these reactions, 500 nM prenylated Ypt31 was loaded onto liposomes by EDTA exchange of GMPPNP at 30 °C for 30 minutes. The presence of GMPPNP in the liposome mixture dictated that Arf1 was added last. Curves were fitted in GraphPad PRISM 10 using a nonlinear regression (one phase association) accounting for drift if apparent in the data to extrapolate the rate constant, which was then divided by the GEF concentration to calculate the exchange rate.

Statistical analysis

All assays were performed in triplicate, and statistical analysis was performed using GraphPad PRISM 10. GEF assay comparisons were analyzed by unpaired parametric t-test. Liposome floatation comparisons were analyzed by paired ratio t-test.

bioRxiv preprint doi: https://doi.org/10.1101/2023.11.22.568272; this version posted November 22, 2023. The copyright holder for this preprint (which was not certified by peer review) is the author/funder, who has granted bioRxiv a license to display the preprint in perpetuity. It is made available under aCC-BY 4.0 International license.

SUPPLEMENTARY REFERENCES

1. B. C. Richardson, C. M. McDonold, J. C. Fromme, The Sec7 Arf-GEF is recruited to the trans-Golgi network by positive feedback. *Dev. Cell* **22**, 799–810 (2012).
2. L. F. Kinman, B. M. Powell, E. D. Zhong, B. Berger, J. H. Davis, Uncovering structural ensembles from single-particle cryo-EM data using cryoDRGN. *Nat. Protoc.* **18**, 319–339 (2023).
3. J. Zivanov, *et al.*, New tools for automated high-resolution cryo-EM structure determination in RELION-3. *Elife* **7** (2018).
4. A. J. Muccini, M. A. Gustafson, J. C. Fromme, Structural basis for activation of Arf1 at the Golgi complex. *Cell Rep.* **40**, 111282 (2022).
5. R. S. Sikorski, P. Hieter, A system of shuttle vectors and yeast host strains designed for efficient manipulation of DNA in Saccharomyces cerevisiae. *Genetics* **122**, 19–27 (1989).
6. P. A. Randazzo, O. Weiss, R. A. Kahn, Preparation of recombinant ADP-ribosylation factor. *Methods Enzymol.* **219**, 362–369 (1992).
7. R. J. Duronio, *et al.*, Protein N-myristoylation in Escherichia coli: reconstitution of a eukaryotic protein modification in bacteria. *Proc. Natl. Acad. Sci. U. S. A.* **87**, 1506–1510 (1990).
8. L. L. Thomas, J. C. Fromme, GTPase cross talk regulates TRAPPII activation of Rab11 homologues during vesicle biogenesis. *J. Cell Biol.* **215**, 499–513 (2016).
9. C. B. Brachmann, *et al.*, Designer deletion strains derived from Saccharomyces cerevisiae S288C: a useful set of strains and plasmids for PCR-mediated gene disruption and other applications. *Yeast* **14**, 115–132 (1998).
10. A. Waterhouse, *et al.*, SWISS-MODEL: homology modelling of protein structures and complexes. *Nucleic Acids Res.* **46**, W296–W303 (2018).
11. E. F. Pettersen, *et al.*, UCSF ChimeraX: Structure visualization for researchers, educators, and developers. *Protein Sci.* **30**, 70–82 (2021).
12. B. C. Richardson, S. L. Halaby, M. A. Gustafson, J. C. Fromme, The Sec7 N-terminal regulatory domains facilitate membrane-proximal activation of the Arf1 GTPase. *Elife* **5** (2016).
13. M. S. Longtine, *et al.*, Additional modules for versatile and economical PCR-based gene deletion and modification in Saccharomyces cerevisiae. *Yeast* **14**, 953–961 (1998).
14. S. Wu, G. J. Letchworth, High efficiency transformation by electroporation of Pichia pastoris pretreated with lithium acetate and dithiothreitol. *Biotechniques* **36**, 152–154 (2004).
15. J. Y. Lee, H. Chen, A. Liu, B. M. Alba, A. C. Lim, Auto-induction of Pichia pastoris AOX1 promoter for membrane protein expression. *Protein Expr. Purif.* **137**, 7–12 (2017).
16. B. C. Richardson, J. C. Fromme, Biochemical methods for studying kinetic regulation of Arf1 activation by Sec7. *Methods Cell Biol.* **130**, 101–126 (2015).
17. S. Q. Zheng, *et al.*, MotionCor2: anisotropic correction of beam-induced motion for improved cryo-electron microscopy. *Nat. Methods* **14**, 331–332 (2017).

bioRxiv preprint doi: https://doi.org/10.1101/2023.11.22.568272; this version posted November 22, 2023. The copyright holder for this preprint (which was not certified by peer review) is the author/funder, who has granted bioRxiv a license to display the preprint in perpetuity. It is made available under aCC-BY 4.0 International license.

18. K. Zhang, Gctf: Real-time CTF determination and correction. *J. Struct. Biol.* **193**, 1–12 (2016).

19. A. Punjani, J. L. Rubinstein, D. J. Fleet, M. A. Brubaker, cryoSPARC: algorithms for rapid unsupervised cryo-EM structure determination. *Nat. Methods* **14**, 290–296 (2017).

20. J. Zivanov, T. Nakane, S. H. W. Scheres, Estimation of high-order aberrations and anisotropic magnification from cryo-EM data sets in RELION-3.1. *IUCrJ* **7**, 253–267 (2020).

21. T. Nakane, D. Kimanius, E. Lindahl, S. H. W. Scheres, Characterisation of molecular motions in cryo-EM single-particle data by multi-body refinement in RELION. *Elife* **7**, e36861 (2018).

22. J. Jumper, *et al.*, Highly accurate protein structure prediction with AlphaFold. *Nature* **596**, 583–589 (2021).

23. P. V. Afonine, *et al.*, Real-space refinement in PHENIX for cryo-EM and crystallography. *Acta Crystallogr D Struct Biol* **74**, 531–544 (2018).

24. D. Liebschner, *et al.*, Macromolecular structure determination using X-rays, neutrons and electrons: recent developments in Phenix. *Acta Crystallogr D Struct Biol* **75**, 861–877 (2019).

25. C. J. Williams, *et al.*, MolProbity: More and better reference data for improved all-atom structure validation. *Protein Sci.* **27**, 293–315 (2018).

26. P. V. Afonine, *et al.*, New tools for the analysis and validation of cryo-EM maps and atomic models. *Acta Crystallogr D Struct Biol* **74**, 814–840 (2018).

27. T. Bepler, *et al.*, Positive-unlabeled convolutional neural networks for particle picking in cryo-electron micrographs. *Nat. Methods* **16**, 1153–1160 (2019).

28. R. W. Klemm, *et al.*, Segregation of sphingolipids and sterols during formation of secretory vesicles at the trans-Golgi network. *J. Cell Biol.* **185**, 601–612 (2009).

29. C. M. Highland, L. L. Thomas, J. C. Fromme, Methods for Studying Membrane-Proximal GAP Activity on Prenylated Rab GTPase Substrates. *Methods Mol. Biol.* **2557**, 507–518 (2023).
